# Street vending, vulnerability and exclusion during the COVID-19 pandemic: the case of Cali, Colombia

**DOI:** 10.1177/09562478221113753

**Published:** 2022-10

**Authors:** Lina Martínez, Graeme Young

**Keywords:** Colombia, COVID-19, global South, informal economy, street vendors

## Abstract

The COVID-19 pandemic has had a profound effect on livelihoods everywhere, but especially in the informal economy where crucial forms of protection and security are often absent. A detailed understanding of the impacts for informal workers, the public policy approaches that could most effectively respond to their needs, and the barriers to such policy, is urgently needed. This paper discusses the results of a 2021 street vendor survey in Cali, Colombia, focusing on (1) vendors’ socioeconomic circumstances and (2) their political engagement and attitudes on key policy and governance issues. It argues that while the pandemic and the government responses to it negatively impacted street vendors, there are steps that government could have taken, and can still take, to address vendors’ needs and priorities. To ensure a just, equitable, sustainable recovery, and to protect economically marginalized groups from future crises, informal workers must be more meaningfully included in decision-making processes.

**Figure:**
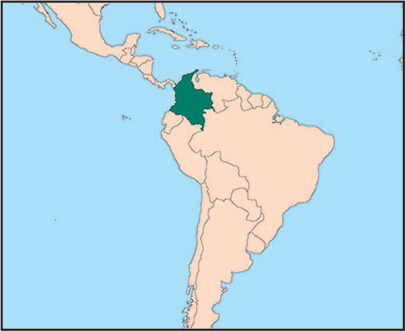


## I. Introduction

Informality is a common characteristic of cities in the global South. In Latin America, an estimated 56 per cent of the working age population earned their income through informal economic activity before the COVID-19 pandemic, with significantly larger shares in extreme cases like Honduras (84 per cent), Guatemala (76 per cent) and Bolivia (71 per cent). The share of employment in the informal economy is expected to increase to about 60 per cent as a result of the significant contraction of the economy in the region.^(1)^ The impact of the COVID-19 crisis on informal workers has been significant. Informal livelihoods are mostly characterized by lower educational attainment, precariousness and the inability of workers to adjust to external shocks,^(2)^ making them particularly vulnerable to the effects of economic crisis. Moreover, countries in the global South do not have the fiscal capacity to provide emergency transfer programmes to replace the lost earnings of a large portion of the informal workforce.^(3)^ Colombia, a middle-income country in Latin America, has experienced one of the most significant periods of economic crisis in its recent history as a result of the pandemic, with unemployment reaching over 20 per cent and poverty rising to 42 per cent in 2020.^(4)^ The quarantine introduced by the government in 2020 lasted for over six months – one of the longest lockdowns in the world – particularly affecting the poorest and informal workers who live hand-to-mouth.^(5)^ This paper contributes to efforts to understand the impact of the COVID-19 pandemic on street vendors, an important segment of informal workers in cities in the global South, and to consider more effective and inclusive policy approaches to informality. Presenting a selection of the results of a survey of street vendors in Cali, the third largest city in Colombia, conducted between March and May 2021, we explore two important issues:

1) **Socioeconomic circumstances**: How the pandemic has affected the livelihoods of street vendors and their families.
2) **Political engagement and policy opinions**: The relationship between government and street vendors during the pandemic, which we analyse by focusing on information about vendors’ trust in institutions, access to public programmes, and how government might address the conditions street vendors face.

We argue that street vendors have been negatively impacted by the pandemic, that government responses have exacerbated their existing vulnerabilities, and that there are clear steps the government could have taken, and can still take, to address vendors’ needs and priorities. Specifically, street vendors experienced work interruptions, income reductions and indebtedness, along with the challenges surrounding educational access for their children. Street vendors also report limited access to public programmes that could potentially serve as a social safety net, protecting them from the effects of the pandemic while also addressing long-standing issues of structural inequality. They also, importantly, point to programmes that they believe the government should focus on, most notably compensatory income and food, and, in lower numbers, to regulations that would allow them to continue to work and to access subsidies for housing. For their needs and priorities to be met, vendors and other informal workers must be included in decision-making processes. Such inclusion is a prerequisite for a just, equitable and sustainable recovery, and is necessary to prevent similar harms in future crises.

As explained below in greater detail, our paper contributes to the literature on street vendors in the global South by expanding on understandings of their socioeconomic conditions during the COVID-19 pandemic, connecting these conditions to their political exclusion, and addressing the shortcomings in policy responses.

The remainder of this paper is organized into a further five sections. The first provides a brief overview of the forms of socioeconomic and political exclusion that street vendors commonly face in cities in the global South. The extent to which these two forms of exclusion are linked is emphasized. Particular attention is given to the potential impact of the COVID-19 pandemic on the power relations that are fundamental to the governance of street vending. The second section provides background information about Cali to offer necessary context for the findings of this study, and the third section outlines the methodology that this study employs. Survey results that are relevant for understanding street vendors’ socioeconomic circumstances and their political engagement and policy opinions are presented in a fourth section. A concluding discussion highlights significant findings and potential policy implications.

## II. Street Vending and Exclusion

Street vendors in cities in the global South have long faced significant socioeconomic challenges. As Roever and Skinner highlight, insecurity, harassment, confiscations and evictions are all common forms of exclusion that have important livelihood effects.^(6)^ The highly public and visible nature of street vending renders it uniquely exposed to the disciplinary and developmental ambitions of the state. While governments might invoke a wide variety of arguments to justify a coercive and restrictive approach to street vending,^(7)^ questions of political and economic power are often central to their actions.^(8)^ Indeed, as Setšabi and Leduka find, apparent concerns about aesthetics and public health can mask a more fundamental desire to support formal businesses and cover policy failures.^(9)^ Street vending, in this sense, is a class issue in which the power of the state is used to enforce certain visions of who and what a city is for and how it should function. In class terms, street vendors are both an apparent manifestation of urban poverty and viewed as a blight on urban aesthetics, an obstacle to vehicular and pedestrian traffic, potentially dangerous, and a threat to formal businesses and government finances.^(10)^ This is true across the global South, including in Latin America, where conflicts surrounding street vending have been well documented.^(11)^ The class dimensions of street vending are compounded by other forms of marginalization that commonly characterize informal work, having to do with gender, migration status and race and ethnicity. These multiple dimensions of structural discrimination create additional barriers, including a lack of access to public services and employment and the difficulty of combining livelihood activities with care work.^(12)^ The state is again an important actor in this regard: in its ability to provide – or fail to provide – forms of support that address ingrained inequalities; its role in shaping migration patterns and denying more straightforward pathways to integration; and in overseeing a political economy in which certain groups and interests are prioritized over others, creating and sustaining the forms of exclusion that intersect in street vending.

This marriage between class power and state power means that the socioeconomic exclusion faced by street vendors is often matched by, and tied to, their political exclusion. Poorly designed or dysfunctional institutions have been widely viewed as a source of exclusion or voluntary exit in the context of informality.^(13)^ While it is difficult to draw a practical distinction between an unwillingness and an inability to operate formally, the fact that both can be viewed as a result of a failure of governance emphasizes the extent to which informal economic activity can be produced by the state.^(14)^ It is not merely the case, however, that the state is failing to adequately perform its core functions, but rather that it embodies and reinforces power structures that systematically favour certain interests over others. For street vendors, this can result in a form of governance that is defined by a combination of hostility and neglect. While street vendors are sometimes able to exert forms of political influence where favourable circumstances exist,^(15)^ three points are worth emphasizing. First, even where the governance of informal economies is comparatively more inclusive, it can nevertheless be incoherent and ineffective,^(16)^ and examples of meaningful inclusion are still hard to identify. Second, as Altamirano finds, individuals in the informal economy may establish weaker links with political parties than others do,^(17)^ suggesting that new approaches to inclusion are clearly needed. Even where opportunities for influence exist, these might be structured around unequal forms of exchange and their benefits might be limited.^(18)^ Finally, and perhaps most significantly here, the fact that opportunities for influence are highly dependent on political circumstances suggests that they can disappear when these circumstances change.^(19)^

The forms of exclusion that characterize street vending predate the COVID-19 pandemic. How, why and to what extent they were compounded by the pandemic, and by government responses to the pandemic, deserves urgent attention. In a major study on the impact of the pandemic on different forms of informal work, the global network WIEGO (Women in Informal Employment: Globalizing and Organizing) highlights the work interruptions and income reductions experienced by street and market vendors in nine of the 11 cities under study. The study identifies problems surrounding demand, supply and price, and other changes related to market closures; the expiration, damage or confiscation of goods; and the destruction of vending sites.^(20)^

The profound effect of the COVID-19 pandemic on governance has also been documented. The rise of technocratic forms of governance can minimize opportunities for democratic decision-making; this is a key way in which the capacity for street vendors to exercise at least some political influence can decline.^(21)^ The pandemic, in certain cases, facilitated the rise of a specific form of technocratic politics that not only, for obvious reasons, prioritized public health over economic issues, but also limited the space in which economic issues could be addressed in the context of a public health crisis. This combination of restricted democratic space and the underlying power relationships characterizing the governance of street vending had significant potential to deepen exclusion and exacerbate existing vulnerabilities. This power relationship often favours a discourse that, as Setšabi and Leduka highlight, prioritizes *“concerns over the lack of order, city aesthetics, public health and apparent social decay and disorganisation”* over *“sustainable livelihoods concerns”*.^(22)^ The pandemic offered an opportunity in which this discourse could flourish in ways that deserve further critical scrutiny.

Colombia offers a particularly interesting case for exploring these themes. This is especially true given that street vendors in the country have, in important ways, been able to assert their rights,^(23)^ and that there has been the possibility for more inclusive forms of governance for informal economic activity. As Swanson notes, *“Bogotá, for instance, has been heralded as a progressive model for modern cities”* as *“[o]ver the last 20 years, city administrators have emphasized the right to the city approach in an effort to promote more inclusionary urban space”.*^(24)^ The extent to which inclusion is realized in practice for street vendors and other marginalized urban groups, is, however, uncertain.^(25)^ More generally, Colombia possesses participatory institutions in which civil society can possibly play important roles,^(26)^ and the country is generally democratic, even if its shortcomings mean that it is classified as a “flawed democracy” in the Economist Intelligence Unit’s (EIU) most recent Democracy Index.^(27)^ In a separate WIEGO report that surveys the impact of the pandemic on street and market vendors in Latin America, the authors claim that the provision of protections by states for informal workers through legislation *“signals a shift in the ways governments address citizens as holders of social rights, which are also justiciable in case of non-compliance by states”*.^(28)^ This does not mean, however, that these efforts have been without their flaws. As the authors note, despite the fact that some states, including Colombia, *“created institutional spaces for enabling social dialogue in the design of public policies to tackle the pandemic, these spaces were largely technocratic and focused solely on the health crisis”*.^(29)^ According to the EIU, Colombia also experienced a democratic decline in 2021, recording its worst score since 2006,^(30)^ and the quality of different aspects of its democracy varies significantly.^(31)^ Studying street vendors in Cali provides an opportunity to examine how the governance of street vending during the pandemic has unfolded in a particular urban context; it also allows us to consider the shortcomings that may have characterized this governance, and the potential for more inclusive forms of governance in the future that might be able to address related forms of socioeconomic exclusion.

Our paper contributes to literature on street vendors in the global South in three ways. First, it adds to understandings of the socioeconomic conditions of street vendors during the COVID-19 pandemic, and, given the importance of understanding these conditions in the context of long-lasting structural forms of exclusion, the vulnerabilities that they face more generally. Second, it connects these socioeconomic conditions with key political questions, highlighting the fundamental connections between socioeconomic exclusion and political exclusion. A post-pandemic recovery must therefore be built on inclusion in decision-making, giving vendors a leading role in identifying their needs and determining how these should be addressed. Rather than adopting a technocratic approach to the issues highlighted here, it is necessary to re-politicize street vending, focusing on the political institutions and processes that govern it and structuring these in ways that empower street vendors and provide space for their meaningful participation in governance. Third, our paper specifically addresses the shortcomings that have characterized policy responses in Cali, and, in doing so, points to alternative directions that new forms of governance could pursue to address street vendors’ needs and, more fundamentally, to ensure that post-pandemic recovery is both inclusive and sustainable. Although we focus on Cali, the need to address the socioeconomic and political exclusion of street vendors across the global South suggests that the insights offered by this case study have potentially broader application.

## III. City Context

With 2.4 million inhabitants, Cali is the third largest city in Colombia.^(32)^ Like other cities in Latin America, it has, over the last few decades, experienced economic growth and an expansion of the middle class.^(33)^ Nevertheless, the city is marked by high inequality, urban poverty and high rates of violence and crime.^(34)^ The city is highly segregated, with the most vulnerable segments of the population living in large communities on its fringes. A large majority of the population lives in neighbourhoods that are characterized by low socioeconomic status, limited access to transport, health facilities and public spaces for recreation, including parks and green spaces, and the highest homicide rates in the city.^(35)^

Informality is a highly visible characteristic of the city. About half of the working age population obtained their income through informal economic activities before the pandemic.^(36)^ Informal workers and businesses in the city conduct diverse activities including transport services, informal vending and small household businesses. The informal sector also hosts migrants, including those displaced by the conflict in the country and immigrants from Venezuela.

In Colombia, the lockdown in response to COVID-19 took effect in mid-March 2020, and lasted for over six months.^(37)^ Street vendors and other informal workers were not permitted to work in the streets during this period. Altogether, the government action paralysed the city’s economic activity, only allowing some essential activities to take place. This shock created the largest economic contraction in the country’s recent history. In 2020, income per capita declined by 8.6 per cent, and monetary poverty^(38)^ rose by about 5 per cent, reaching 42 per cent of the population. Extreme poverty increased over the same year, moving from 9.6 per cent to 15.1 per cent.^(39)^ To alleviate the economic consequences of the lockdown, the government created a cash transfer programme called *ingreso solidario* (solidarity income), transferring money to over four million households in the country during the pandemic, starting in March 2020 and extending through 2022. The subsidy, which varied according to the number of residents in a household and levels of poverty, ranged from US$ 116 (for one person) to $ 152 for a household of five.^(40)^

The pandemic deepened the vulnerabilities of informal workers and increased the size of the informal sector, particularly as the unemployment rate rose to 23 per cent in 2020, a 10 percentage point increase over 2019. The local government estimated that by 2021, the working age population in the informal sector could increase to 60 per cent (from 50 per cent before the pandemic) as a consequence of the economic setback resulting from COVID-19.^(41)^

**Map 1:**
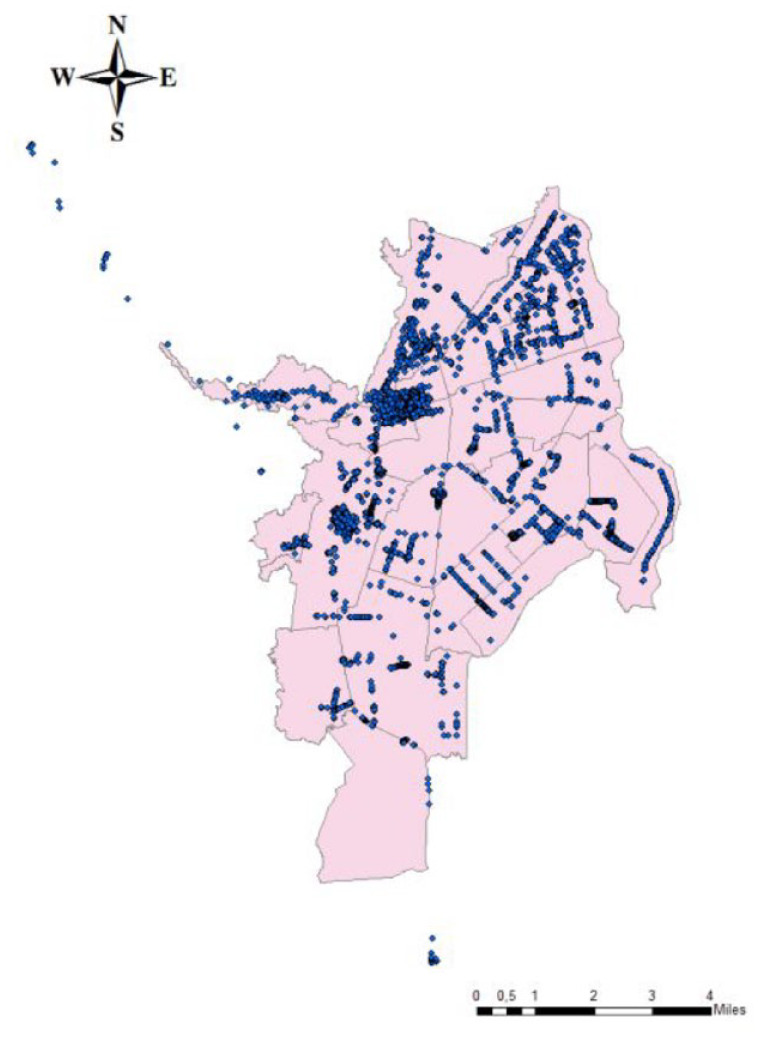
Street vendors’ distribution across the city, 2019 Source: POLIS 2020.

Street vending is one of the main occupations in Cali’s informal sector. In 2019, the local government counted 10,280 street vendors located across the city, with the largest concentration in neighbourhoods with a low socioeconomic status. Downtown, a 13-block area in the city centre, located alongside the bus stations, holds the main spatial cluster of informal sales in the city. Food markets also host a large concentration of street vendors, as do locations with high levels of formal commerce. Map 1 presents the distribution of street vendors in the city, with each dot representing one vendor.

## IV. Data and Methods

The primary data for this analysis come from a phone survey of 750 street vendors collected between March and May 2021. The research was funded by UK Research and Innovation through the Centre for Sustainable, Healthy and Learning Cities and Neighbourhoods (SHLC), and is part of a broader project that aims to promote inclusive governance for street vendors in Cali. This study relied on the assistance of leaders of street vendors’ organizations, who were contacted to explain the purpose of the project and the need for a survey on street vendors’ experiences during the pandemic and their perception of government response to the crisis. These leaders subsequently reached out to members of their associations to ask them to participate in the survey. Seventy per cent of the participants belonged to an informal workers’ association, and the majority worked in the Downtown area, where the two largest street vendors’ associations in the city have affiliates.

Street vendors who voluntarily agreed to participate were contacted by trained research assistants to take part in the phone survey. The interview took about 25 minutes to complete, although some took considerably longer due to interruptions as vendors conducted their businesses. At the end of the interview, research assistants asked informants for referrals to potential participants. The majority of study participants were contacted in this way. Vendors were asked about their demographic characteristics (age, gender and race), family composition, educational access for their children during the pandemic, economic activity, reductions in income, access to financial services, their trust in institutions and relationship with the government, and their health. To guarantee anonymity, no personal information (names, addresses or identification numbers) was collected. The survey was approved by the Universidad Icesi ethics committee (code number 348). The data of this study, data protocols and complete questionnaire are published and available through open access.^(42)^

Table 1 presents the demographic data of survey participants. Just under half of participants were women, the average age was 51 years old, over 33 per cent belonged to racial/ethnic minority groups, and 73 per cent identified as the household head (the primary household income earner).

**Table 1:** Demographic characteristics of street vendors

Women (%)	49
Average age (years)	51
Race: minority (%)	33
Race: non-minority (%)	67
Household head (%)	73

The following section presents a descriptive analysis of the most relevant survey results for understanding two issues: 1) the socioeconomic consequences of the pandemic for street vendors; and 2) street vendors’ political participation and relations with government during the crisis. Differences by gender and race/ethnicity, and mean differences between groups using a T-test (Stata 15) are shown to establish statistical values.

## V. Results

The results on vendors’ socioeconomic conditions during the pandemic focus on their income and working days, their household composition and children’s education and their levels of indebtedness. We then explore political engagement and policy opinions, focusing on vendors’ levels of trust in different institutions, their access to public programmes and their views on the programmes the government should prioritize. Taken together, these two sets of issues provide invaluable insights into the needs and priorities of street vendors and point to ways that these can be addressed.

### a. Socioeconomic conditions of street vendors during COVID-19

Past research on street vendors in Cali shows at least three patterns.^(43)^ First, street vendors are a highly vulnerable group. Most are characterized by lower educational attainment – having completed only elementary school with a few years in secondary education. Few participate in the welfare system, and the large majority are household breadwinners providing for two to three children.^(44)^ Second, informal sales in the city are highly fragmented. Street vendors are a diverse group, and they vary by sales location and the type of goods being sold. Vendors located in food markets are, generally speaking, the most vulnerable. They have lower levels of educational attainment and are disproportionately members of minority groups and displaced persons. Vendors located downtown, in the historic centre and the largest settlement in the city, have, on average, higher levels of education and of participation in welfare programmes.^(45)^ Third, street vendors earn on average about 20 per cent above the average monthly minimum wage.^(46)^ However, like other informal workers, they are unable to capitalize on their relatively higher incomes because of their exclusion from formal financial systems. To access credit, the only option for most informal workers is to resort to payday lenders who charge predatory interest rates of over 20 per cent per month.^(47)^

Past research has also shown that street vendors need to work almost every day to cover their basic expenses, such as food, rent and transportation.^(48)^ Every day that they are off the streets represents a day without the means to cover these basic needs. The COVID-19 pandemic represented a significant impediment in this regard, creating the risk that many of them would fall into extreme poverty as a result.

#### Income and working days during the pandemic

Before the pandemic, street vendors reported working on average for 10 hours a day, six days a week, earning about $    400^(49)^ a month.^(50)^ Our survey suggests that the pandemic had a significant effect on their ability to work (see Table 2). A large majority (over 70 per cent) of our sample reported being unable to work for more than three months, and when they were able to work, they were on the streets for about six days a week for nine hours a day. A higher proportion of street vendors belonging to minority groups reported not being able to work for more than three months. On top of the heavy restrictions imposed by the quarantine, when street vendors could work they experienced a large decline in sales and income, particularly women and members of minority groups; women earned on average 15 per cent less than men, and members of minority groups earned about 22 per cent less than non-minority workers. Average monthly sales during the pandemic (other than lockdown times) were reported to be $ 369 and their profit (minus merchandise costs and transport) was reported to average $ 145. While, as stated above, street vendors earned, on average, 20 per cent over the minimum wage in 2017,^(51)^ during the pandemic, their monthly income declined to 48 per cent of the minimum wage.^(52)^

**Table 2:** Economic conditions during the pandemic

	Total	Male	Female	Diff.	Minority	Non-minority	Diff.
*Average days without working during the pandemic (%)*							
Less than a month (30 days)	4	4	3		1	5	***
Between one and two months (30–60 days)	22	24	20		17	26	***
More than 3 months (+90 days)	74	72	77		82	69	***
No loss of working days (0 days)	0	0	1		0	0	
*After lockdown*							
Average days per week working	5.8	5.8	5.8		5.8	5.8	
Average hours worked per day	9.1	9.7	9.1	***	9.5	9.4	
Average monthly sales (USD)	369	412	353	***	324	412	***
Average monthly profit (USD)	145	171	132	***	135	162	**

* 1 US$ (USD) = 3,400 Colombian pesos (average currency – 2021).

*** *p* > 0.99, **p > 0.95.

**Figure 1:**
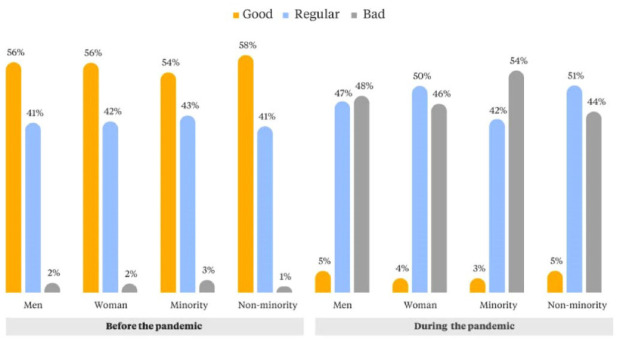
Perceptions of income adequacy

Ninety-eight per cent of street vendors surveyed in this study reported experiencing a reduction in their income during the pandemic. The income reduction for street vendors has been larger than that of other informal workers. An online survey collected in Cali in 2020 showed that 59 per cent of informal workers surveyed declared that their income was “good”.^(53)^ When questioned about their perception of their income before COVID-19, over 50 per cent of our participating men and women considered that it had been good (enough to cover basic needs and to save). Similar results are reported by past research, in which over 60 per cent of street vendors in the city reported that vending provided enough resources to meet their needs.^(54)^ During the pandemic, most workers declared that their income was either “regular” (enough to cover only basic needs) or “bad” (not enough to cover basic needs) (Figure 1). There are no notable differences by gender, but members of minority groups also reported a reduction in income during the pandemic.

#### Household composition and children’s education

On average, informal workers reported living with four people in their household, two of whom were their children. Workers belonging to minority groups reported more household members and a slightly higher average number of children than non-minority workers. Forty per cent of street vendors have school-age children, and 22 per cent of these workers reported that their children dropped out of the educational system during the pandemic.

**Figure 2:**
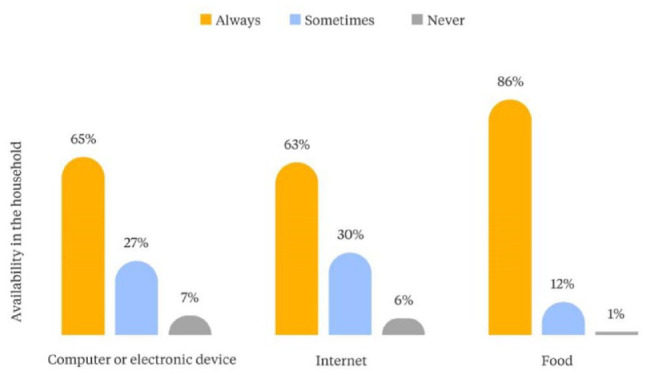
Availability of computer/electronic device, internet and food in the household during the pandemic

As shown in Figure 2, fewer than 65 per cent of street vendors always have an electronic device or internet connection available to support online education for their children; another 27–30 per cent sometimes have this access. The power dynamics that can exist within households and that shape the internal distribution of resources, however, mean that access is not necessarily equal. The inability to provide the resources for online classes may have contributed to the high rates of dropout reported by street vendors. Another concerning finding relates to food security. Only 86 per cent of street vendors reported always having food in the household (Figure 2). Moreover, 21 per cent of street vendors declared that someone in the household went to bed hungry each night during the pandemic, a figure that was significantly higher amongst members of minority groups (27 per cent). We could surmise that the dropout rate may also be a result of the perceived need for children to work to support the provision of basic household needs, or of their inability to focus on school.

**Table 3:** Household composition

	Total	Male	Female	Diff.	Minority	Non-minority	Diff.
Average number of people living in the household	3.6	3.5	3.7		3.8	3.5	*
Average number of children	2.1	2.2	2.1		2.4	2.1	**
Households having children of school age (%)	40	40	39		38	41	
Children dropped out of school during pandemic (%)	22	22	23		21	23	
Someone in the household went to bed hungry during the pandemic (%)	21	21	21		27	19	**

*** *p* > 0.99, ** *p* > 0.95, * *p* > 0.90.

**Table 4:** Indebtedness during the pandemic

	Total	Male	Female	Diff.	Minority	Non-minority	Diff.
Before the pandemic you had debts or loans (%)	33	32	33	_	30	36	_
During the pandemic you applied for a loan (%)	21	23	20	_	18	23	_
Loan with family (%)	19	17	20	_	24	16	_
Loan with friends (%)	19	21	16	_	14	18	_
Loan with a bank (%)	24	26	22	_	21	27	_
Loan with payday lender (%)	29	30	28	_	36	29	_
Loan with micro-finance institution (%)	5	5	5	_	2	5	_
Amount last loan (USD)	290	332	264	_	249	328	_
Amount last instalment (USD)	30	31	33	_	28	33	_
Have difficulties paying debts	77	77	78	_	85	74	_

* 1 US$ (USD) = 3,400 Colombian peso (average currency – 2021)

None of the differences reached significance.

#### Indebtedness

Informal vending is an activity that is highly dependent on cash flows. Street vendors need money in their pockets to be able to buy goods and work every day. They have very few options to access regulated financial markets. Given their high vulnerability, employment instability and lack of records to certify their income, their only option to access credit and cash are their closest networks and payday lenders.^(55)^

Before the pandemic, over 30 per cent of street vendors were in debt. Most likely, the debt was incurred from a payday lender, since over 50 per cent of loans acquired by street vendors (before the pandemic) were accessed through this unregulated service.^(56)^ Street vendors had used their loans for multiple purposes: the large majority (55 per cent) used their loan to invest in their business (buying merchandise); 38 per cent used it to pay rent; 34 per cent to pay utilities; and 31 per cent to pay debts.

During the pandemic, over 20 per cent of street vendors needed to access credit, most commonly from a payday lender (29 per cent), followed by a bank (24 per cent), or family or friends (each at 19 per cent). The average amount of the credit was about equal to the monthly minimum wage ($298). The last credit payment was about $30, meaning that street vendors have, on average, about $115 dollars per month to provide for a household of four.

### b. Political engagement and policy opinions

Before the pandemic, the informal sector in Cali was experiencing a transformation in its regulatory framework.^(57)^ The major transformation of the regulatory framework to protect workers’ rights was introduced in Colombia in 1991. That year, the country approved a constitutional reform and introduced an instrument called *tutela* (guardianship). *Tutelas* are used by citizens to claim immediate protection of fundamental rights. Since the reform’s introduction in 1991, *tutelas* have become an efficient and cheap mechanism through which citizens could claim the protection of fundamental rights ranging from their right to health access to the right to work.^(58)^*Tutelas* allow any citizen, without the intermediation of a lawyer, to assert their constitutional rights in the event that these are violated (for instance, through the denial of health access or prohibitions against working in a public space), and these appeals are required to be reviewed by a judge within 10 days. If a judge favours the citizen’s petition, government institutions are obligated to respond to the needs of the citizen, for instance, providing health access or impeding the police or any local authority from removing a worker from public space.

This instrument provided informal workers requiring public space in Colombia with the ability to claim their fundamental right to work. In 2003, after the review of several *tutelas* filed by street vendors in Bogotá, the Constitutional Court ruled that the eviction of street vendors from public space should be prohibited, since the eviction from public space was a violation of the right to work (Constitutional Court ruling 772-2003). This ruling also required that if local government planned to evict informal workers from public space, they must first provide those who would be affected by permanent eviction with equal or better income-generating opportunities elsewhere.^(59)^ This ruling, as a consequence of several *tutelas* filed in Bogotá, became a countrywide precedent in the regulation of public space and is the precedent for the most recent national policy regulating street vending (Law 1988). This was enacted in 2019, and for the first time requires local governments to balance the general use of public space with the economic requirements of street vendors, recognizing their right to work in public space.^(60)^

The national policy framework to regulate street sales, which had been in discussion since 2019 (Law 1988), generated tensions between informal workers and governments at the local and national level. Local authorities in Cali have long used policing, evictions and fines as measures to control informal sales in public space (even though this is prohibited by the ruling from the national *tutela* 772-2003), generating conflict and tensions with informal workers, and with very little success in reducing the growth of informal sales because street vendors return to public space shortly after they are evicted.^(61)^ The new national policy also contradicts the intentions of the local authorities which aim at *“recuperation of the public space”* by removing informal traders.^(62)^ Since the beginning of the pandemic in 2020, there have been no reports of local authorities planning extensive evictions or use of public space regulations.

The relationship between informal workers and local authorities in Cali has been defined by distrust given the extent of the repressive measures taken in the city to harass, evict or fine workers in public space.^(63)^ During the pandemic, in addition to significant impoverishment, informal workers also experienced low levels of political engagement; almost 80 per cent of respondents in our sample declared they felt unsupported by the government during the crisis and only 23 per cent reported being the beneficiary of any subsidy and/or benefit promoted by the state. Additionally, 22 per cent of street vendors declared that police harassment had increased during the crisis, although 60 per cent stated they were not victims of harassment by the police.

In order to explore vendors’ political participation and perceptions of government support during the pandemic, our survey included a set of questions about their trust in government institutions, their access to public programmes, the programmes street vendors considered most relevant during this crisis, the levels of harassment they were subjected to and their participation in workers’ organizations.

Table 5 presents the average scores on selected questions pertaining to governance. On a scale of 0–10, in which 0 means no trust and 10 complete trust, street vendors rated their trust in the city council, the police and civil servants, at about 4 points. Women, on average, had more trust in the police than did men. Scores of trust in government institutions amongst survey participants are, generally speaking, similar to the scores amongst the general population. In 2018, Cali residents rated their trust in public institutions at about 3.2 (combining the trust scores relating to the city council, the police and the civil servants), about one point lower than informal workers did at the time.^(64)^

**Table 5:** Institutional trust and government support

	Total	Male	Female	Diff.	Minority	Non-minority	Diff.
*Institutional trust (on a scale of 0–10)*							
Municipal Council	3.9	3.9	4.0		3.9	4.0	
Police	4.1	3.8	4.3	**	4.1	4.1	
Civil service	4.1	4.2	4.1		4.3	4.1	
*Access to public programmes*							
Job placement (%)	0	0	1		0	0	
Education and new skills (%)	1	1	1		1	1	
Unemployment insurance (%)	1	2	1		1	2	
Public housing (%)	3	3	3		3	3	
Cash transfers (%)	18	15	22	**	22	18	
School access for children of street vendors (%)	39	39	39		37	44	
*What programmes should the government prioritize?*							
Finance inclusion (%)	26	27	25		21	28	**
Work training (%)	30	30	29		26	32	
Education (%)	24	23	24		24	24	
Relocation (%)	14	13	15		14	14	
Subsidies for housing (%)	46	48	45		49	46	
Compensatory income – cash transfer (%)	64	65	64		67	65	
Food (%)	61	66	55	***	65	62	
Regulations to allow informal workers to continue working in the public space (%)	48	48	49		43	49	

*** *p* > 0.99, **p > 0.95.

The participation of street vendors in publicly funded programmes is low. Three per cent declared they had access to housing subsidies and 18 per cent had access to national cash transfers, although figures for the latter were higher for women and members of minority groups. A majority of street vendors believe that during the crisis government should have been focused on compensatory income (64 per cent) and food assistance (61 per cent); followed by regulations to allow them to work in the public space and subsidies for affordable housing (48 per cent and 46 per cent respectively).

As stated above, 70 per cent of our respondents reported belonging to an informal workers’ association, a figure that is possibly high as a result of the methods employed for contacting respondents. Our survey also suggests, however, that membership is not necessarily a pathway to political inclusion; only 39 per cent of vendors surveyed believed that belonging to an association increased political participation. This is perhaps due to the nature of associations in Cali, which allow members to get together and discuss common issues and access information from association leaders, but, beyond a limited number of cases in which small programmes may exist, do not provide members with direct benefits.

## VI. Discussion and Policy Implications

The survey results presented here offer several important insights into the lives and livelihoods of street vendors during the COVID-19 pandemic. Four points have particular potential to inform public policy design and implementation, and are highlighted here for discussion.

First, the pandemic has had a clear negative impact on the livelihoods of street vendors in Cali. Street vending is a precarious livelihood activity, a reality exposed and made worse by reduced incomes, work interruptions and indebtedness. The experiences of street vendors in Cali had much in common in this regard with those of informal workers more generally around the world during the pandemic.^(65)^ An average income of about $ 115 per month for a family of four represents a significant constraint on the ability of vendors to meet their household needs. Without targeted interventions to provide street vendors with the support they require, there is little reason to believe that their circumstances will improve.

This difficulty is compounded by the second important challenge that is worth highlighting here, which is the fact that street vendors have limited access to programmes from which they could potentially benefit. Only a small percentage of respondents have access to job placement, education and skills development, unemployment insurance or public housing programmes, and although more have access to cash transfers – and, most notably, affordable quality education for their children – these rates are still low. If street vendors require targeted forms of state support, it is clear that those that exist are inadequate.

Our survey also offers useful insights into the types of support that street vendors believe the government should prioritize. Of the eight options included in the survey, two enjoy majority support: compensatory income and food. Two more, improved regulations and subsidies for housing, are supported by a large minority of respondents. Few vendors expressed support for relocation. This points to a third important insight: the fact that it is crucial to understand and address the different dimensions of economic exclusion that affect street vendors. Some vendors might want formal jobs, while others may prefer forms of support that allow them to continue to engage in their current activities. Street vendors must not be treated as a monolithic group. Their needs and desires may diverge, and governments must have the capacity and flexibility to respond to whatever forms these might take.

Finally, and perhaps most crucially, if governments wish to provide meaningful support for street vendors and address multidimensional forms of economic exclusion, they must work with them to establish priorities and include them in policy design and implementation.^(66)^ A failure to do so will not only perpetuate the disempowerment of an already marginalized group, it will also preclude the creation of effective policies. The responses to our survey suggest that there is much work to be done given clear problems with street vendors’ trust in institutions and a sense of a lack of support. They also suggest, however, that levels of trust amongst vendors are not exceptionally low, particularly compared to the very similar levels of trust recently reported by other city residents – a fact that perhaps indicates broader failures in governance – although the extent to which trust may have been impacted by the major protests in the country that began in 2021, centred on Cali, is uncertain. If street vendors are to trust governing institutions, they must believe they can rely on and benefit from them. Government at all levels must prove this is the case.

What street vendors therefore need is a new social contract, one that cements institutionalized forms of participation, provides basic rights and delivers collectively defined benefits. The forms of exclusion that vendors have faced during the pandemic have exacerbated existing vulnerabilities, but it is important to recognize that these are linked to the long-standing reality of structural exclusion that is the result of a combination of state power and class power. The pandemic has highlighted the negative consequences that exclusion from governance can have for street vendors and, more broadly, the dangers of a technocratic politics that does not provide adequate space for democratic decision-making on important economic issues. The contours of socioeconomic inclusion, especially where exclusion can take a variety of forms and spur multiple needs and interests, can only be determined in processes of negotiation that provide space for representation and voice. This, of course, is the essence of politics, highlighting the extent to which it is necessary to re-politicize street vending and move beyond top-down and technocratic forms of governance and development, even where these are well meaning, in favour of processes that are more participatory, deliberative and democratic.

The exact forms that this might take, in Cali or elsewhere, deserves further consideration. As the successful use of *tutelas* suggests, the law can be an important mechanism through which street vendors can assert their rights. A note of caution is necessary, however, as the law is so often a source of marginalization for street vendors. Indeed, informal livelihoods, by definition, take place at least in some ways outside of the formal legal and regulatory boundaries for economic activity that are established and enforced by the state. Reforming laws that structurally exclude informal workers is vital for empowerment, suggesting that informal workers should not only be able to benefit from laws, but also participate in their design.^(67)^

There is also much work to be done in order to realise the potential for informal workers to organize and to collectively define and assert their rights. While WIEGO’s call for “Nothing For Us, Without Us” focuses on the involvement of leaders of vendors’ organizations in policymaking processes,^(68)^ our survey suggests that informal workers’ associations in Cali are not, at present, necessarily vehicles for agency and empowerment. This failure may point to organizational or strategic shortcomings but might also simply reflect that these groups are forced to operate in an environment in which opportunities to exercise influence are limited. Whether it is a cause or a consequence of a form of technocratic politics in which a combination of state power and class power defines the exclusion of street vendors, it is clear that a void exists around representation that needs to be filled. Addressing this could be key to promoting inclusive governance.

The possibility that new institutional arrangements could have significant benefits should also be recognized. More open participatory processes, built around permanent institutionalized deliberative bodies, localised forms of governance and the involvement of grassroots organizations and movements in ways that are non-hierarchical, could also be valuable. The forms of exclusion that street vendors face were not caused by the pandemic, but a post-pandemic recovery can, and indeed must, nevertheless establish a strong foundation of inclusion. Only then can it be truly just, equitable and sustainable.

## Footnotes

1. Bentala et al. (2020).

2. Chen (2012).

3. See reference 1.

4. DANE (2020, 2021b).

5. Taylor (2020).

6. Roever and Skinner (2016).

7. For arguments for and against street vending, see Bromley (2000).

8. Setšabi and Leduka (2008); Young (2017).

9. Setšabi and Leduka (2008).

10. Young (2017), page 725.

11. For different case studies of street vending in Latin America, see Crossa (2009, 2016); Donovan (2008); Mackie et al. (2014); Steel (2012); Swanson (2007).

12. For different forms of marginalization that exist in informal work, see Kabeer et al. (2013).

13. Berens (2020); de Soto (1989); Perry et al. (2007).

14. For the role of the state in producing informal economic activity, see Young (2019).

15. Cross (1998); Gillespie (2017); Holland (2017).

16. Young (2020).

17. Altamirano (2019).

18. Cross (1998); Holland (2017).

19. Young (2017, 2018).

20. Chen et al. (2021).

21. Young (2017).

22. Setšabi and Leduka (2008), page 239.

23. Meneses-Reyes and Caballero-Juárez (2014).

24. Swanson (2018), page 359.

25. See reference 24, pages 355–363.

26. Mayka (2019).

27. Economist Intelligence (2022), page 49.

28. Marchiori and Assis (2021), page 21.

29. See reference 28, page 22.

30. See reference 27, page 33.

31. See reference 27, page 49.

32. DANE (2018).

33. Araujo et al. (2014).

34. Martínez (2017).

35. Martínez (2021).

36. DANE (2015).

37. See reference 5.

38. In 2021, the monetary poverty line in Colombia was set at 354,000 Colombian pesos – about US $104 dollars (estimated at $1 = 3,400 Colombian pesos). Extreme monetary poverty was set at 161,099 Colombian pesos, corresponding to $47. This is the poverty line per person for a monthly income.

39. DANE (2021a).

40. El Comercio (2021).

41. El País (2021).

42. Martínez et al. (2022).

43. Martinez and Estrada (2017); Martínez and Rivera-Acevedo (2018); Martínez et al. (2017, 2018).

44. Martínez and Short (2017).

45. Martínez et al. (2017).

46. See reference 45.

47. Martínez and Rivera-Acevedo (2018).

48. Saha (2011).

49. This estimation was made in 2018 based on an exchange rate of $ 1 = 2,000 Colombian pesos. In 2021, given the devaluation of the Colombian peso, the exchange rate is estimated at $ 1 = 3,400 Colombian pesos.

50. Martínez et al. (2018).

51. See reference 47.

52. The Colombian minimum wage in 2021 is $ 298 dollars per month ($ 1 = 3,400 Colombian pesos).

53. POLIS (2021).

54. See reference 50.

55. See reference 47.

56. See reference 47.

57. Brown et al. (forthcoming).

58. Carrera Silva (2011).

59. Constitutional Court (2003).

60. Law 1988 (2019).

61. Semana (2019).

62. Alcaldía de Cali (2017).

63. See reference 44.

64. Martínez et al. (2020).

65. See reference 20.

66. Also see reference 28.

67. For an influential analysis of informality and the law in Latin America, see de Soto (1989).

68. See reference 20, page 34.
